# Exploring Italian Autochthonous *Punica granatum* L. Accessions: Pomological, Physicochemical, and Aromatic Investigations

**DOI:** 10.3390/plants13182558

**Published:** 2024-09-12

**Authors:** Deborah Beghè, Martina Cirlini, Elisa Beneventi, Chiara Dall’Asta, Ilaria Marchioni, Raffaella Petruccelli

**Affiliations:** 1 Economics and Management Department, University of Parma, Via J.F. Kennedy 6, 43125 Parma, Italy; 2 Food and Drug Department, University of Parma, Parco Area delle Scienze, 27/A, 43124 Parma, Italy; elisa.beneventi@gmail.com (E.B.); chiara.dallasta@unipr.it (C.D.); ilaria.marchioni@unipr.it (I.M.); 3 Institute of BioEconomy, National Research Council (CNR-IBE), Via Madonna del Piano n. 10, Sesto Fiorentino, 50019 Florence, Italy; raffaella.petruccelli@ibe.cnr.it

**Keywords:** pomegranate, qualitative analysis, ancient cultivars, local products, volatile profile, HS-SPME/GS–MS

## Abstract

Autochthonous Italian pomegranate accessions are still underexplored, although they could be an important resource for fresh consumption, processing, and nutraceutical uses. Therefore, it is necessary to characterize the local germplasm to identify genotypes with desirable traits. In this study, six old Italian pomegranate landraces and a commercial cultivar (Dente di Cavallo) were investigated, evaluating their fruit pomological parameters, physicochemical (TSS, pH, TA, and color) characteristics, sugar content, and aromatic profiles (HeadSpace Solid-Phase MicroExtraction (HS-SPME)) coupled with Gas Chromatographyass Spectrometry (GC–MS) of pomegranate juices. Significant differences were observed in the size and weight of the seed and fruits (127.50–525.1 g), as well as the sugar content (100–133.6 gL^−1^), the sweetness (12.9–17.6 °Brix), and the aroma profiles. Over 56 volatile compounds, predominantly alcohols (56%), aldehydes (24%), and terpenes (9%), were simultaneously quantified. Large variability among the genotypes was also statistically confirmed. The results indicate a strong potential for commercial exploitation of this germplasm, both as fresh and processed fruit, and highlight its versatility for diverse applications. The genetic diversity of the autochthonous pomegranate accessions represents a precious heritage to be preserved and enhanced. This work represents a preliminary step toward a more comprehensive characterization and qualitative valorization of the Italian pomegranate germplasm.

## 1. Introduction

Pomegranate (*Punica granatum* L.) is one of the world’s oldest domesticated fruit crops, and its cultivation is considered to have started in the Neolithic Age [1]. The species has a long history of diffusion, initially grown in the region between the Caspian Sea, the Caucasus, and Northern Turkey and later spreading westward to Northern Africa and the Mediterranean Basin [2,3]. Pomegranate has a rich genetic patrimony, with over 500 described cultivars and a large number of wild plants. However, its germplasm has been only partially explored so far [4].

Despite the wide genetic diversity, only 50 cultivars have been characterized and widely grown so far. This poor understanding and preservation may therefore lead to a drastic loss of the existing biodiversity [4,5].

In the last decade, however, this species has gained considerable attention due to its nutritional properties and significant health benefits, which are closely linked to the fruit’s rich content of nutrients and bioactive compounds; indeed, a well-ripened pomegranate fruit shows a good amount of protein (1.6%), carbohydrate (14.5%), and fibers (5.1%), as well as mineral elements (e.g., phosphorous: 70 mg/100 g; magnesium: 12 mg/100 g; and calcium: 10 mg/100 g) and antioxidant compounds. The latter includes ascorbic acid (20 mg/100 g) and (poly)phenolic compounds, mainly represented by organic acids and flavonoids (including flavonoids, anthocyanins, proanthocyanidins, ellagitannins, and gallotannins). Their amount is highly dependent on the intrinsic pomological features of each variety/accession (e.g., color) [6].

This unique and complex phytochemical composition is deemed responsible for a wide range of health-promoting biological activities [7].

Consumed as fresh seeds, juice, and various processed products, pomegranate also holds substantial market potential as an ingredient in food supplements, functional foods, and herbal products. 

Because the demand for high-quality, locally produced food is particularly high in the current market, a comprehensive characterization of the autochthonous pomegranate genotypes may offer opportunities for innovative product design.

This is particularly compelling in Italy, where the market still heavily relies on international cultivars, despite the increase in cultivated areas (from 36 to 2000 ha in the last 15 years [8]) and the large market opportunities. In response to the growing interest in this crop, several programs have been launched to conserve, identify, and study autochthonous genotypes from different regions across the Italian peninsula. Several collections have been established, in particular, in Sicily, Latium, Basilicata and Campania, Apulia, and Emilia Romagna [4,9,10,11].

Local pomegranate accessions, widespread only in restricted geographical areas, could be actually reintroduced into cultivation, helping to improve the diversity and sustainability of local production and at the same time preserving traditional agricultural systems. Pomegranate is particularly suitable for this purpose, because it can be grown in marginal areas that are not suitable for conventional agricultural cultivation due to their bioclimatic and socioeconomic characteristics. In recent years, various strategies have been implemented for the valorization of these areas, including their re-cultivation with emerging fruit crops like fig, pomegranate, and pistachio, which have low agronomic demands. Therefore, increasing local production would reduce the need for imports, thus supporting local economies and enhancing both agricultural resilience and biodiversity [12].

However, in order to boost and promote Italian pomegranate production, more in-formation is needed on the morphological, chemical, and biochemical characteristics of the different local genotypes, as the overall quality of pomegranate fruits is highly dependent on their nutritional and organoleptic profile. A full compositional and sensorial characterization will help in identifying those cultivars that align with consumer preferences, allowing for selecting, cultivating, harvesting, and marketing fruits with desirable attributes [13,14].

This research, part of a multidisciplinary project aimed at enhancing lesser-known cultivars and their local production, aims at studying the genotypes of Italian pomegranates from pomological, physicochemical, and aromatic perspectives. The results, although preliminary, will contribute to the assessment of biodiversity, support future breeding efforts, and identify high-quality accessions to expand the potential of local production.

## 2. Results and Discussion

### 2.1. Pomological Analysis of Pomegranate Accessions

In this study, seven Italian pomegranate genotypes (Table 1) were analyzed for the morpho-pomological characters of the fruit and seeds, each of them harvested in its environmental niche. Such a unique combination of genetic background and environmental conditions resulted in a large variability in quantitative traits, as presented in Table 2. The size of the fruit and the color of the epicarp are key quality parameters in the international market for fresh products. Consumers particularly appreciate ‘large’ or ‘very large’ fruits. Colorful fruits are also preferred, especially those referred to as ‘red’ [15,16]. The accessions studied showed a fruit skin color ranging from reddish-yellow (three accessions) to red-purple (one accession) (Table 1). The mean fruit weight was 327.2 g among the accessions, comparable to the weight of the fruits of many pomegranate cultivars [9,17,18,19]. 

According to the descriptors used by Bellini et al. [16], the AP2 and BA1 accessions can mainly be classified into the category of ‘very large’ fruits (>350 g); the cultivar DC also falls into this class. Instead, the fruits of the accessions LI1, TU1, and TU2 can be categorized into ‘medium/large’ fruits (150–350 g). According to Tarantino et al. [11], who report the classification of the Codex Alimentarius Commission, the AP2 accession could be categorized in A (>500 g), while BA1 and TU1 could be in the B (401–500 g) and C (301–400 g) classes, respectively. Regarding the equatorial fruit diameter and length, accession AP2 recorded the highest values, 9.06 cm and 10.08 cm, respectively, comparable to the average values observed in the Dente di Cavallo (DC) cultivar. Accession TU3, instead, showed the smallest fruit size compared to others, with values of 6.08 cm and 6.49 cm for FD and FL2, respectively (Table 2). 

All the accessions as well as the DC cultivar showed fruit with a closed or semi-closed calyx (Table 1). However, minimal differences were observed between the accessions for the length, diameter, and shape of the calyx. Similar to the other fruit morphological traits, the AP2 accession showed the highest values for CL and CD (1.90 cm and 2.69 cm, respectively), while the TU3 accession showed the lowest values (Table 2). The results are in the ranges reported by other authors for cultivars and/or local accessions [10,17,18,19].

The ratio between the equatorial diameter and length without the calyx of the fruit ranged from 0.76 to 1.19 for all the accessions; the LI1 and DC samples had the lowest FSI value, whereas TU1 had the highest (Table 2). This ratio defines the shape of the fruit. Three fruit shapes (circular, circular to angular, and angular) have been established by UPOV (2013) [20], while four forms (oblata, rounded-spheroid, ellipsoid, and ovoid) were identified by Bellini et al. [16]. Descriptors of DUS (Distinctiveness, Uniformity, and Stability) for fruit shape (round, ratio <1.0–1.1; ovate, 1.1–1.2; oval, 1.2–1.3; and elliptical, (>1.3) have been used to describe twelve cultivars of pomegranate [21]. The results showed that of all the accessions had the fruit of a round and an ovate shape (Table 1). 

In agreement with the data collected for fruit weight, the highest peel and carpel membrane weight values were observed in AP2 and BA1, followed by the DC cultivar; this parameter represents the inedible part of the fruit and constitutes waste for consumers and industry. The fruit skin and carpellary membrane weight is inversely correlated to the seed weight in each fruit, and therefore, the accessions showed different proportions between the edible and inedible parts; for the STW parameter, the highest values were measured in the DC cultivar (240.8 g) and in the AP2 accession (210.2 g), while lower weights were recorded for the other local accessions. However, in terms of seed yield, calculated as the percentage of seeds (FW − SCW)/FW × 100, it ranged from 40% to 57%, with AP2 having a lower seed yield (about 40%) compared to the other accessions: LI (44%), TU1 (45%), BA (46%), TU2 (48%), TU3 (57%), and DC (55%). These results are comparable to those reported in the study on six cultivars of global commercial interest [22]. As underlined by the authors, the final fruit size or weight is not an indicator of higher yields in seeds. Another important parameter is juice yield (%), which was determined as the quantity of juice (ml) obtained by manual extraction from 20 g of seeds divided by 20 g × 100. In fact, the yield of juice ranged from 65.9% (DC) to 73.3% (BA1).

In addition to the weight of the seeds, which affects the fresh weight of the fruit and its processing, another parameter associated with commercial quality is seed size. Regarding seed characteristics, the samples showed an average seed size with a mean length (SL) of 0.95 cm and a mean seed diameter (SD) of 0.71 cm. The AP2 accession had the highest values for both parameters (SL, 1.09 cm; SD, 0.88 cm), comparable to those of the DC cultivar. According to the DUS and/or UPOV criteria and considering the SL parameter, the seeds fell into two categories: the accessions LI1, TU2, TU3, and BA1 had short seeds (<10 mm), while the TU1 and AP2 accessions and DC cultivar had medium seeds (10–15 mm). In relation to the SD parameter, the seeds of all the samples fell into the medium category (5–7.5 mm). According to Khadivi et al. [23], the seed shape was prismatic in the AP2, BA1, and DC samples, while the others were ovoid in shape. The results of this study agree with Adiletta et al. [10] who reported, in local accessions of the Campania region (Italy), average values of 10.3 mm and 7.9 mm for the seed length and width, respectively, which are lower than the sizes reported by Ferrara et al. [24]; the authors, evaluating 13 pomegranate accessions, typical of Apulia, registered values from 9.8 mm to 14.6 mm and 7.2 and 14.6 mm for the seed length and width, respectively. The morphological parameters of the seeds are shown in Table 3. The results highlighted statistically significant differences between all the samples (*p* ≤ 0.05). Statistically significant differences between the genotypes were also recorded for the seed weight with values ranging from 0.22 g for LI1 to 0.51 g in AP2. AP2 showed a mean value per seed close to the value observed in DC of 0.50 g (Table 3). Cristofori et al. [25] reported weights between 0.28 g and 0.36 g in Italian autochthonous cultivars, while previous studies have reported wide weight ranges per seed in Spanish and Italian cultivars with values in the range of 24 mg and 51 mg [11,24,26]. Moreover, recently, mean values of 0.15–0.52 g (mean 0.36 g) per seed were detected in fourteen pomegranate genotypes collected in Morocco [27]. 

Fruit skin thickness is another important parameter affecting the commercial market quality of pomegranate, as it clearly affects fruit cracking and the storage ability as well as transportation and packaging [19,28]. Its mean value was 0.31 cm, ranging from 0.42 cm (LI1) to 0.27 cm (DC) (Table 2). The fruit skin thickness (FT, mm) was found to be non-significant among all the samples, because the values were very variable in fruits of the same accession due to the difficult detection of this parameter.

### 2.2. Physicochemical Parameters in Juice 

The selected parameters recorded for the juice obtained from the edible part of the fruit (seed) are reported in Table 4, along with their statistical analysis.

The titratable acidity (TA) was significantly different (*p* ≤ 0.05) among the accessions. The lowest average value was obtained in the Dente di Cavallo cultivar and AP2 accession (TA, 0.37% and 0.50%, respectively), while LI1 showed significantly higher titratable acidity (3.96%) compared to the other samples. The TA and pH of a juice can be correlated with its acidity; in particular, the TA, which measures the total concentration of organic acids (expressed as % citric acid), can contribute to the sensorial profile of pomegranate juice and is often used to classify the pomegranate cultivars. Sweet cultivars have a TA value below 0.9%, while cultivars with values between 1 and 2% are classified as sweet–sour [29]. According to this classification, the majority of studied accessions can be classified as sweet–sour, except AP2 and DC (sweet), and are therefore preferably intended for processing.

Regarding pH, the accessions AP2 and BA1 showed the highest pH values, 4.53 and 4.24, respectively. The other accessions had pH values comparable to those of the Dente di Cavallo cultivar (Table 4). The range of pH values determined in this study was similar to those obtained by other authors for both commercial cultivars [29,30] and local germplasm [31,32,33]; pH values have been reported from 2.56 to 4.3 and from 2.8 to 3.8 for cultivars grown in Spain [29] and in Turkey [30], respectively. Regarding the local pomegranate germplasm, Barone et al. [31] have detected values from 3.08 to 4.0 in Sicilian genotypes, as well as by Cirillo et al. [32] in local Campanian accessions. However, all the accessions showed average values higher than the range reported by the US Food and Drug Administration (pH range 2.93–3.20) [29]. 

The taste and quality of pomegranate seeds and the juice also depend on the total soluble solids (TSS) parameter, which can indicate the level of sweetness. The TSS values ranged from 12.9 °Brix for BA1 to 17.6 for DC. Although all the local accessions had lower values compared to the commercial cultivar Dente di Cavallo, the recorded TSS value was higher than 12%, a threshold considered as acceptable for commercial use [34]. The TSS content determined in the accessions of this study was very similar to commercial pomegranate cultivars grown in Italy (15–18 °Brix) [22] and Turkey (15–17 °Brix) [30] and/or for local Italian genotypes analyzed by other authors, such as Ferrara et al. [17] in Apulian genotypes (15–18 °Brix), Barone et al. [31] in Sicilian cultivars (12.9–16.8 °Brix), and Cristofori et al. [25] in five pomegranate accessions collected in Viterbo (Lazio) (12.9–17.8 °Brix). 

The TSS parameter, associated with acidity, was used by Melgarejo et al. [35] to classify pomegranate cultivars in relation to sweetness. The TSS and its TA ratio (TSS/TA) are the main indices for determining fruit quality. In particular, the TSS/TA ratio is an indicator of flavor, quality, and maturity [36]. The studied accessions showed a value between 44.5 and 4.9, with the highest value determined in the Dente di Cavallo cultivar (Table 4). Martinez et al. [37] differentiated Spanish cultivars into acidic, sweet–sour, and sweet using the TSS/TA ratio. The accessions, according to the above classification, fall into the group of acidic cultivars, except AP2 and DC, which are classified as sweet. 

Finally, juice color is an important characteristic that influences consumers’ acceptance and perceived quality. The color parameters of the juices showed significant differences across the genotypes (Table 4). The L value ranged from 36.2 to 15.1; the highest was found in the accession TU2, while BA1 showed the lowest values. The a* color coordinate, which indicates the intensity of the red color of the juice, was highest in the accessions TU1 and TU2, 29.6 and 26.9, respectively, while BA1 showed the lowest values (Table 4). The accession BA1 also showed lower yellowness (b* parameter) compared to the other samples. All the analyzed accessions had higher a* and b* values than the Dente di Cavallo cultivar. The analyzed juices showed a color ranging from light red to red, comparable to the color observed in Algerian cultivars that showed variation in juice color from a light red to dark red color [38]. 

### 2.3. Juice Sugars Characterization 

The amounts of individual soluble sugars and total sugars in the pomegranate accessions are reported in Table 5. The total sugar content, which is the sum of individual sugars, showed an average concentration of 114.8 g L^−1^. Among the accessions studied, BA1 had the lowest content at 100.0 g L^−1^, while TU1 and AP2 had the highest concentrations of total sugar, 133.6 and 131.6 g L^−1^, respectively (Table 5). Fructose and glucose were the predominant sugars, representing 52% and 45% of the total, respectively, according to previous studies [39,40,41]. Generally, the results indicated that the juices from different samples had similar amounts of glucose and fructose. TU1 and AP2 presented the highest glucose levels, while the other accessions, including the DC cultivar, had average values of around 48 g L^−1^ with minimal differences between them. A similar trend was observed for the fructose concentration. The ranges of glucose and fructose detected in our samples were similar to those reported in other cultivars: recently, Arlotta et al. [13] reported glucose values between 43.18 and 64.16 g L^−1^ and fructose values between 29.89 and 68.90 g L^−1^ in local Sicilian pomegranate cultivars. Additionally, our study detected the presence of galactose and mannitol (Table 5). Small traces of galactose were found in almost all the samples except for the TU3 accession, with levels close to 0.5 g L^−1^. Significant quantities of mannitol, a sugar alcohol, already described in pomegranate juice [42], were also present in all the samples, with concentrations ranging from 2.60 g L^−1^ in TU1 to 3.09 g L^−1^ in TU3.

Finally, the glucose/fructose ratio ranged from 0.63 to 0.97. These results are in accordance with the glucose-to-fructose ratio reported in previous studies, i.e., Mena et al. [43] reported that in pomegranate cultivars grown in Spain, the glucose/fructose ratio ranged from 0.88 to 0.96. Considering the different sweetness power of fructose and glucose, their ratio may affect the overall juice taste and therefore the fruit’s sensorial quality. 

### 2.4. Characterization of the Volatile Profile of Pomegranate Juices

The overall quality of pomegranate juices depends on their taste components, aroma profiles, and color properties. Among these factors, aroma is a crucial quality criterion that significantly influences consumer acceptance and preference. 

**Figure 1:**
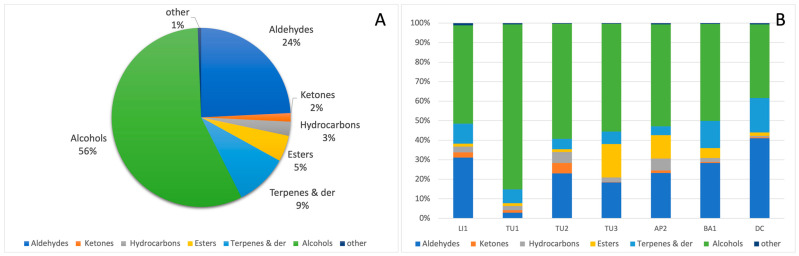
The percentage compositions of the volatile chemical groups found in the total juice pomegranate obtained by all the accessions together (**A**) and in the juice of separate accessions (**B**). For interpretation of the references to color in this figure legend, the reader is referred to the web version of this article.

**Figure 2:**
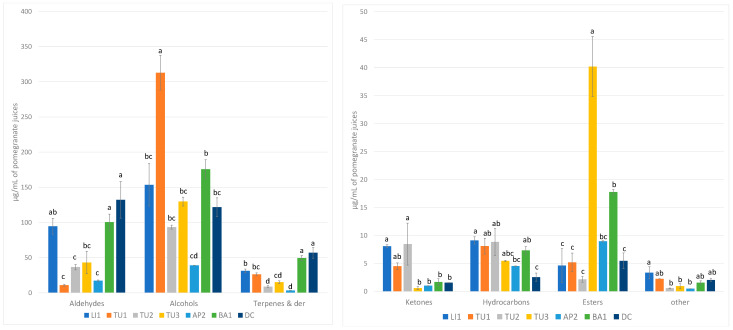
The amounts (μg/mL) of the volatile chemical groups found in the juice of each pomegranate accession. The different letters above each column indicate significant differences among the samples (Tukey’s test *p* ≤ 0.05). For interpretation of the references to color in this figure legend, the reader is referred to the web version of this article.

The volatile profiles of local Italian pomegranate juices were analyzed using HS-SPME/GC-MS. A total of 56 volatile compounds were identified and semi-quantified through internal standard addition, as detailed in Supplementary Table S1. In particular, 15 aldehydes, 13 terpenes and derivatives, nine alcohols, seven esters, six hydrocarbons, six ketones, and one ether were found. The volatile fraction of the pomegranate juice samples that resulted was indeed mainly composed, on average, of alcohols (38–85%), aldehydes (3–41%), and terpenes (5–18%), which is consistent with prior studies on pomegranate juice, in which the same classes of volatiles are reported as predominant [44]. For example, in the previous study on the volatile characterization of Italian and Montenegrine pomegranate juice, Beghè et al. [44] described amounts of alcohols ranging between 34% and 46%, aldehydes between 20% and 34%, and terpenes between 8% and 27%, depending on the pomegranate ecotype considered. Moreover, lower amounts of esters (6%), hydrocarbons (3%), ketones (2%), and other compounds (1%) were measured in all the samples (Figure 1A). Because 4-methyl-benzadehyde and acetophenone presented a co-elution, they were measured together and were considered as ‘other’ in the total volatile quantity, with caprylic ether the only ether detected in all the considered samples. Nonetheless, significant differences in the volatile profiles were observed among the various samples, indicated in Figure 1B and Figure 2.

Alcohols were the dominant chemical group both quantitatively and qualitatively in all the samples, followed by the aldehydes group (except for TU1, where the terpenes and derivatives group was detected in higher amounts) and then the terpenes group (except for TU3, where the esters were detected in higher amounts). The commercial cultivar DC was indeed the sample in which alcohols presented the lower percentage (about 38%), with the aldehydes group (40%) representing the major class. 

Interestingly, the TU1 juices showed the highest alcohol content and, in the same samples, the lowest aldehydes and terpenes amount in respect to all the other analyzed samples. 

Regarding alcohols, the detected range was from 98.00 µg/mL for AP2 to 312.88 µg/mL for TU1. 1-Hexanol, with characteristic herbal aromatic notes, was the predominant compound in all the juices analyzed with amounts ranging between 24.63 and 228.21 µg mL, followed by (Z)-3-hexen-1-ol found in the range 8.42–69.20 µg/mL, which is associated with green and leafy flavors. The relevance of these two compounds is consistent with previous studies conducted on pomegranate juice [44,45]. Beghè et al. [44] reported concentrations of 1-hexanol that ranged between 8.95 ± 0.27 and 168.44 ± 5.86 µg/mL and quantities of (Z)-3-hexen-1-ol ranged from 5.29 ± 0.07 to 42.82 ± 5.40 µg/mL among pomegranate juices derived from fruits belonging to different ecotypes. Similarly, high percentages of these two compounds (21–44% of 1-hexanol, and 8.6–18% of (Z)-3-hexen-1-ol) were found in pomegranate juices obtained from five different accessions cultivated in Turkey [45]. These two components contributed to differentiating samples; in particular, the quantity of 1-hexanol was significantly higher in the TU1 and TU3 samples and lower in the DC cultivar, whereas (Z)-3-hexen-1-ol was significantly higher in LI1, TU1, and TU3 compared to AP2 and DC. 

Aldehydes constituted the second most abundant volatile group, ranging from 17.3 µg/mL for AP2 to 132.20 µg/mL for DC. Aldehydes were one of the most representative chemical classes in the overall volatile fraction of the analyzed pomegranate juices, as indicated in previous studies, in which aldehydes represented about 20–34% of the pomegranate juice volatile fraction [44]. Among aldehydes, the most abundant was hexanal, with the exception of TU1, in which it was almost absent. Hexanal, with herbal notes, was the most quantitatively abundant compound, although not present in all the samples, ranging from 0 µg/mL for TU1 juice to 101.00 µg/mL for DC, followed by 2-hexenal, associated with a sweet flavor, absent only in TU1 juice and detected at a maximum level of 22.32 µg/mL in LI1 juice. 

Hexanal, together with other volatile compounds such as the already mentioned 1-hexanol, (Z)-3-hexen-1-ol, and some terpenes (β-pinene, limonene, α-terpineol, and β-caryophyllene), is considered of fundamental importance in the definition of pomegranate juice aroma [46]. 

Other important aromatic compounds present in the juice samples were terpenes and derivatives, including β-Myrcene, limonene, Eucalyptol, γ-Terpinene, o-Cymene, β-linalool, β-caryophyllene, terpinen-4-ol, menthol, alpha-terpineol, p-cymen-8-ol, and geranyl acetone, as well as other unidentified terpenic compounds. In particular, limonene ranged from 0.96 µg/mL for AP2 to 18.71 µg/mL for DC, and β-caryophyllene, present only in three samples, showed higher values in BA1 (15.78 µg/mL) and DC (12.12 µg/mL). These were followed by α-terpineol and terpene ni, ranging from 1.17 µg mL and 0 for AP2 to 7.51 µg mL and 5.31 µg mL for DC, respectively.

Besides α-terpineol, β-linalool, p-cymen-8-ol, and menthol were also reported to be detected in minor levels in the different pomegranate juices. In particular, β-linalool was detected in concentrations of 0.57 ± 0.22 µg/mL–2.18 ± 0.20 µg/mL and *p*-cymen-8-ol in amounts of 0.37 ± 0.09 µg/mL–0.56 ± 0.06 µg/mL, while in a previous study conducted on pomegranate juice derived from other Italian accessions, these components presented values of 0.65 ± 0.00 µg/mL–6.13 ± 0.16 µg/mL and 0.16 ± 0.23 µg/mL–0.58 ± 0.07 µg/mL, respectively [44]. The same compounds were also reported in a Turkish pomegranate juice but in lower amounts in respect to those observed in the current study (about 0.002 µg/mL) [47]. Menthol was present only in two juice samples prepared from fruits harvested in the Tuscany region. Similar amounts of this volatile compound (0.96 ± 0.65 µg/mL) were observed in a previous study in pomegranate juice derived from Italian cultivars, while higher amounts were observed in Montenegrine juice samples (0.36 ± 0.51 µg/mL–3.56 ± 0.12 µg/mL) [44]. High proportions of limonene were found in almost all the considered samples. Limonene, along with other terpenes like β-myrcene, α-terpineol, and β-caryophyllene, was found to be the most abundant in the pomegranate juice volatile fraction [9,45,48]. Limonene, with a citrus aroma, was found to be the most representative terpene in the LI, BA, and DC samples. In these latter juices, β-caryophyllene, associated with sweet and woody notes, presented the highest concentrations, while in all the other samples this component was almost absent. 

All the other components, belonging to the ketone, ester, and hydrocarbon classes, were detected at minor levels in the considered pomegranate juices. Among the ketones, 2-nonanone, with fruity notes, was the compound measured in higher amounts in all the samples, as reported also by Güler and Gül and Catania et al. [45,49]. Esters, generally responsible for fruity flavors, presented a different distribution among the considered samples; in particular, isoamyl acetate was found in high amounts in TU3, AP2, and BA1, while in the other juices, lower concentrations were detected. Other consistent esters found in all the analyzed samples were ethyl caprylate, ethyl caprate, and methyl salicylate, already found in other research studies [49,50,51]. 

With regard to the esters group (isoamyl acetate, ethyl caproate, hexyl acetate, ethyl caprylate, ethyl caprate, methyl salicylate, and ethyl laureate), the concentration varied from 2.14 µg/mL, for TU2, to 40.20 µg/mL, for TU3. In the present study, isoamyl acetate was detected in high amounts compared to other components belonging to the esters group; however, it was found only in the TU3, BA1, and AP2 juices. 

Ketones and hydrocarbons were detected in the juices only at minor levels: from 0.58 µg/mL for TU3 to 8.46 µg/mL for TU2 (ketones group) and from 2.56 for DC to 9.12 µg/mL to LI1 (hydrocarbons group). 2-nonanone was the most prevalent compound in the ketones group with a concentration that varied from 0 for TU3 to 4.1 µg mL for TU2, while styrene, with sweet balsam and floral sensory properties (concentration from 0 for LI and DC to 7.4 µg/mL for BA1), was the most prevalent in the hydrocarbon group.

### 2.5. Principal Component Analysis

In order to better assess the potential association between the pomegranate samples and the parameters measured within this study, a Principal Component Analysis (PCA) was conducted using pomological, physicochemical, sugar, and volatile profiles. Concerning volatiles, compounds occurring only in one sample have been excluded. 

The total variance explained by the first three principal components (PCs) in the model was 70.12%; a plot of the percentage of variance explained by seven PCs was reported in the Supplementary Material (Figure S1). The first principal component PC1 explained the 29.12% total variance. A large majority of the volatile compounds, including most terpenes and their derivatives, aldehydes, and alcohols, showed a positive loading on this component, while styrene, isoamyl acetate, and menthol showed a negative loading on PC1. For the physicochemical parameters, the TSS showed a positive loading, whereas the color parameter (L and *b) showed a negative loading. 

Only two pomological descriptors (FSI and CSI) showed a negative loading on PC1. On the contrary, a large majority of the morphological descriptors showed a significant loading on PC2, which explains 22.01% of the total variability. The physicochemical parameters TA and TSS/TA index showed a high loading. 

PC3, explaining 19.05% of the total variance, showed a positive loading with 1-nonanol (fresh, fatty, and floral), heptanal (green and fatty), octanal (aldehydic), nonanal (waxy), β-linalool (floral), 1-octanol (waxy, green), caprylic ether, phenyl ethyl alcohol (floral), and β-caryophyllene (woody), for the volatile compounds, and CD and SSI for the morphological descriptors. Sugar showed a significant loading on PC3.

**Figure 3:**
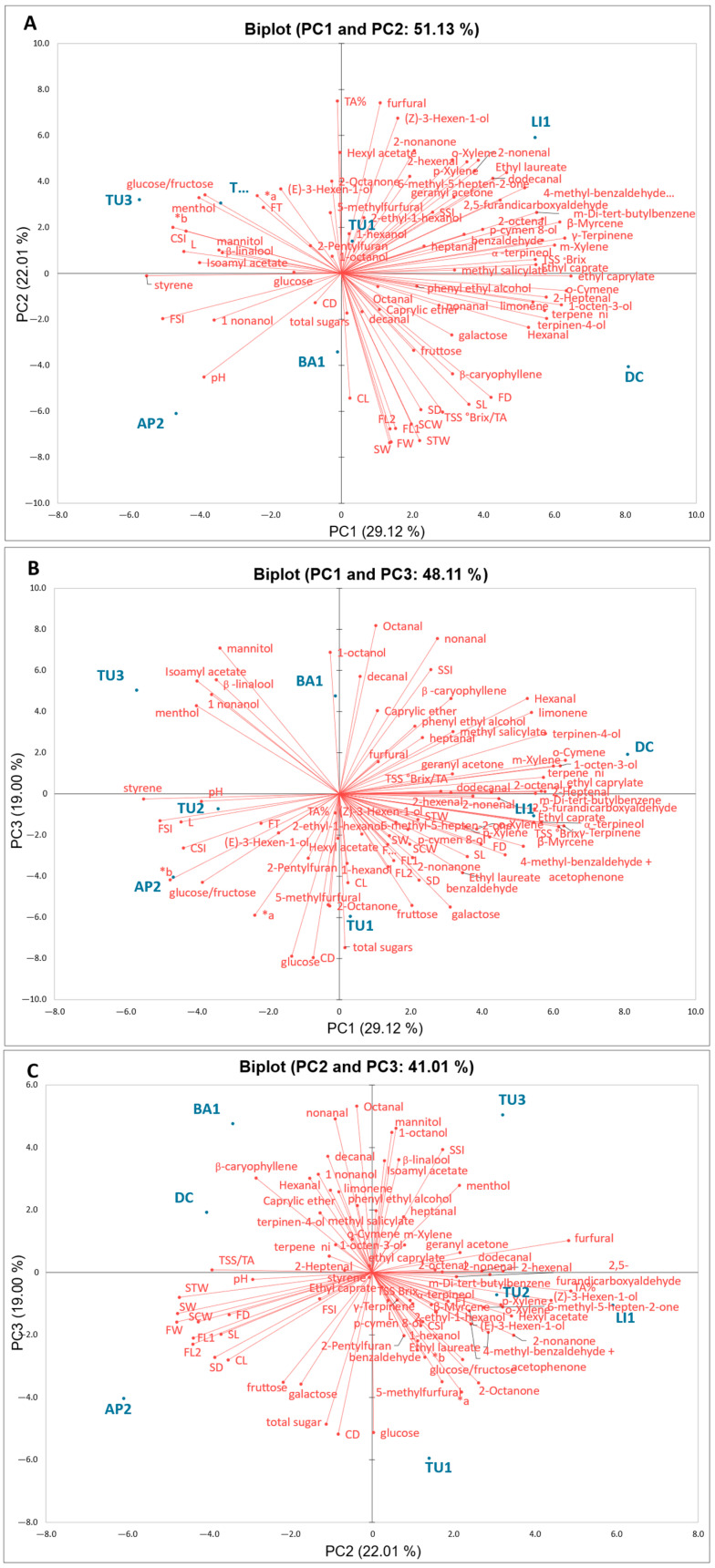
(**A**–**C**) The loading plots of the first, second, and third principal components showing the positions of the pomegranate accessions and the different parameters studied.

The PCA score plot (Figure 3) showed a good association of pomegranate samples according to their characteristics. Focusing on the PC1 and PC2 plot, the accessions were grouped by PC1 based on the volatile classes and some of the physicochemical parameters of their juices. In particular, DC and LI1 are located in the positive region of PC1 and were characterized by more aldehydes and terpenes compared to the other juices, TU2, TU3 and AP2. These classes of volatile compounds are mainly associated with sweet, green, floral, and fruity notes. So, it is reasonable to assume that these pomegranate juices presented a more pronounced aroma in respect to the other considered samples. Moreover, TU2, TU3, and AP2 in PC1 stood out from the others for their greater red coloration and brightness (a* and L color parameters). The samples varied significantly in PC2 according to their increase in size and weight per fruit and seeds; AP2, DC, and BA1, located in the positive region of PC2, have fruits and seeds of a larger size compared to TU1, TU2, TU3, and LI1. This is consistent with the literature [32], which reports that pomegranate samples from different geographical origins exhibit high variability in all analyzed traits, including differences in average fruit weight and overall fruit size. 

## 3. Materials and Methods

### 3.1. Plant Material 

This study was conducted on six old genotypes of pomegranate (*P. granatum* L.), landraces, sampled in different Italian regions compared with a cultivar of commercial interest, Dente di Cavallo (DC). The genotypes were cultivated in Liguria (LI1), Tuscany (TU1; TU2; and TU3), Apulia (AP2), and Basilicata (BA1), while the one commercial cultivar, Dente di Cavallo (DC), was in the Sicily region. 

The names of the accessions and codifications are reported in Table 1 and the pictures of their fruits are shown in Figure S2. The fruits were harvested at maturity based on a color index, which requires the ground color to be uniform across the entire epicarp surface. Harvesting occurred from September to November 2019, depending on the ripening period in different regions, and the fruits were then transported to the laboratory under controlled temperature conditions, in an air-conditioned vehicle. Upon arrival, they were processed immediately to ensure the preservation of their quality. The fruits were harvested and processed within 24–36 h depending on the distance of the harvesting location to the laboratory.

The samples were prepared according to Beghè et al. [9] for the morphological analysis and Beghè et al. [44] for the juice analysis, respectively. Briefly, for each accession, 5 fruits of uniform size were harvested around the canopy. All the seeds were extracted manually from each fruit. A total of 60 seeds from each fruit were randomly selected for the morphological analysis that was conducted before and after the removal of the sarcotesta by hand. The remaining seeds were utilized for the juice preparation. The juice of each pomegranate was obtained by placing the arils on a metal sieve and manually gently pressing them. Then, a subsample of the mixed juice of five fruits was put into individual conical tubes of 15 mL (Falcon), filtered and stored, after passage in liquid nitrogen, and then kept frozen at −80 °C until analysis. 

### 3.2. Pomological Characterization

The fruits and seeds of the different genotypes were subjected to morphological measurements according to the description provided by Beghè et al. [9], which included the length (mm), width (mm), shape (length/width ratio), and weight (g). The measurements comprised the following: (a) fruit characteristics: total fruit fresh weight (FW, g); fruit equatorial diameter (FD, cm); fruit length without calyx (FL1, cm); total fruit length (FL2, cm); calyx equatorial diameter (CD, cm); calyx length (CL, cm); fruit skin thickness equatorial (FT, mm); fruit skin and carpellary membranes weight (SCW, g); fruit shape index (FSI); calyx shape index (CSI); (b) seed characteristics: total seeds weight (STW, g); seed weight (SW, g); seed length (SL, cm); and seed diameter (SD, cm). Linear dimensions (length, width, and thickness) were determined with a caliper with ±0.01 mm accuracy, and the weight was measured using a precision balance accurate to 0.001 g. The morphological characters evaluated included 15 quantitative traits. Furthermore, some qualitative characters (e.g., color of epicarp and fruit shape) were observed; these traits are listed in Table 1.

### 3.3. Physicochemical Parameters in Juice

The total soluble solids (TSS), pH, titratable acidity (TA), and color were detected in pomegranate juices. The TSS was measured using a digital refractometer (HannaHI96811; HANNA Instruments, Italy) and expressed in °Brix; the pH values were determined using a digital pH meter (Crison Basic 20, Barcelona, Spain) calibrated with pH 4 and 7 buffers; and the TA was determined according to the AOAC method 22.060 [52] and expressed in %. Based on the values of the TSS and TA, the Maturity Index values (IM) were calculated as a TSS/TA ratio. The juice color was measured in the CIELab system using a Minolta CR-200 chromatometer (Minolta, Ramsey, NJ, USA), determining L (brightness; 0 = black and 100 = white), a* (red = positive values and green = negative values), and b* (yellow = positive values and blue = negative values) values. The analyses of the physicochemical parameters were performed in triplicate.

### 3.4. Juice Sugars Characterization 

The carbohydrate contents were analyzed by High-Performance Liquid Chromatography according to Deslauriers et al. [53], with modifications. Briefly, 5 mL of freeze pomegranate juice was filtered through muslin cloth and the juice without a solution buffer was centrifuged for 5 min at 10,000× *g* at 4 °C, and the supernatant was filtered through a 0.20 µm filter (Millex-FG; Merck Millipore Co., Darmstadt, Germany) and diluted 1:1 with ultra-pure water. The resulting extract was then used for assessing the carbohydrates using a PerkinElmer-HPLC system (Series 200 Flexar HPLC, PerkinElmer) equipped with a refractive index detector (LC-30 RI, PerkinElmer, Waltham, MA, USA) and a Shodex Sugar SC 1011 column (8 mm × 300 mm; Showa Denko GmbH, Munich, Germany), maintained at 88 ± 1 °C, which was preceded by a pre-column Sugar-Pak II Guard-Pak Insert (Waters, Milford, MA, USA). The mobile phase was water, Milli Q grade (Burlington, MA, USA), at 0.5 mL min^–1^. The sample injection volume was 5 µL. The identification and quantification of individual carbohydrates was carried out by a comparison of the retention times with those of authentic carbohydrate standards (Sigma-Aldrich Italia, Milan, Italy) [54]. The analyses of the soluble carbohydrates were performed in triplicate.

### 3.5. Volatile Fraction Characterization

The chemical composition of the pomegranate juice sample volatile fraction was performed by extracting analytes by means of the headspace solid-phase micro-extraction technique (HS-SPME) and analyzing them on a chromatography-mass spectrometer (GC-MS). In particular, a tri-phasic fiber coated with 50/30 μm of Divinylbenzene–Carboxen–Polydimethylsiloxane (DVB/Carboxen/PDMS; Supelco, Bellefonte, PA, USA) was used, and the analyses were performed on a gas chromatograph (Thermo Scientific Trace 1300, Waltham, MA, USA) and a single quadrupole mass spectrometer (Thermo Scientific ISQ), with an electronic impact (EI) source. The instrument was equipped with a SUPELCOWAX 10 capillary column (Supelco, Bellefonte, PA, USA; 30 m × 0.25 mm × 0.25 μm). All the parameters applied for the analyte extraction, separation, detection, and identification were the same as those described in Beghè et al. [44]. All the compounds of interest were semi-quantified on the basis of the use of a reference (Toluene).

### 3.6. Statistical Analysis

All the results are expressed as the mean ± standard error. The data were compared using one-way Analysis of Variance (ANOVA) followed by a Tukey post hoc test at a 95% confidence level. The dataset underwent a Principal Component Analysis (PCA) to better assess the potential association among the samples and measured parameters. The analysis was performed using XLSTAT v. 2023 software (Addinsoft, New York, NY, USA).

## 4. Conclusions

This study evaluated the pomological, physicochemical, and aromatic characteristics of six pomegranate landraces and the cultivar Dente di Cavallo (DC) from different regions in Italy. Significant differences were observed among the pomegranate genotypes for many of the investigated parameters. Notably, the fruit weight (FW) ranged from 127.50 g (TU3) to 525.1 g (AP2), total soluble solids (TSS) ranged from 12.9 °Brix (BA1) to 17.6 °Brix (DC), titratable acidity (TA) ranged from 0.50% (AP2) to 3.96% (LI), and sugar content ranged from 100 g/L (TU3) to 133.6 g/L (TU1). In terms of the aromatic profiles, the juice samples were primarily composed of alcohols (38–85%, AP2-TU1), aldehydes (3–41%, TU3-DC), and terpenes (5–18%, AP2-DC). 

The significant differences observed indicate that these accessions are a valuable resource for genetic improvement and the enhancement of local products. The results suggest a strong potential for the commercial exploitation of this germplasm, both as fresh and processed fruit. For example, accession AP2 showed favorable pomological and physicochemical characteristics (e.g., big fruit and seed size, sweetness, and high pH), making it suitable for the fresh use of the fruit. However, other accessions, like TU3, showed characteristics such as the small size of the fruits and seeds but a high seed yield, making them more suitable for juice production. 

Although the current results are only preliminary and do not allow for separating the effect of the environment from the genetic background, all the accessions showed different features that make them a unique and valuable source of biodiversity. 

This multidisciplinary approach allowed for obtaining an initial but comprehensive overview of the peculiarities of the various genotypes, providing valuable information for the enhancement and conservation of local genetic resources. 

The results obtained can also contribute to improving selection and cultivation strategies, promoting sustainability and the quality of fruit production. 

In conclusion, the accessions studied could be used for new production, cultivation in marginal environments, or for breeding purposes. 

Further studies will be conducted to evaluate the content and presence of nutraceutical compounds that could be used in pharmaceutical, medical, and/or cosmetic applications.

## Figures and Tables

**Table 1:** The accession code (ID), accession name, province (region, country), and fruit main qualitative morphological parameters of pomegranate genotypes used in this study.

ID	Accession Name	Province (Region, Country)	Fruit Shape	Epicarp Color	Shape of Base	Shape of Apex	Type of Calyx
LI1	Melograno di La Spezia	La Spezia (Liguria, Italy)	Rounded	Red-purple	Rounded-open	Rounded-necked	Semi-open
TU1	Melograno di Firenze	Firenze (Tuscany, Italy)	Ovate	Reddish-yellow	Rounded-angular	Rounded-necked	Semi-open
TU2	Melograno di Buti	Pisa (Tuscany, Italy)	Rounded	Medium red	Truncate	Rounded-necked	Semi-open
TU3	Melograno di Lucca	Lucca (Tuscany, Italy)	Rounded	Orange red	Rounded-angular	Truncate-necked	Closed
AP2	Tardiva di Puglia	Lecce (Apulia, Italy)	Ovate	Reddish-yellow	Rounded-open	Truncate-necked	Semi-open
BA1	Melograno di Matera	Matera (Basilicata, Italy)	Ovate	Medium red	Rounded-angular	Rounded-necked	Semi-open
DC	Dente di Cavallo	Catania (Sicily, Italy)	Rounded	Reddish-yellow	Rounded	Rounded-necked	Closed

**Table 2:** Pomological characters of pomegranate genotypes. FW: fruit weight; FD: equatorial diameter; CL: calyx length; CD: calyx diameter; FL1: fruit length without calyx; FL2: total fruit length; FT: fruit skin thickness equatorial; SCW: fruit skin and carpellary membrane weight; FSI: fruit shape index; CSI: calyx shape index.

ID	FW (g)	FD (cm)	CL (cm)	CD (cm)	FL1 (cm)	FL2 (cm)	FT (cm)	SCW	FSI	CSI
LI1	179.2 ±22.4 ^d^	7.74 ± 0.8 ^b^	1.40 ± 0.43 ^ab^	2.32 ±0.58 ^ab^	5.86 ± 0.23 ^b^	7.26 ± 0.38 ^b^	0.42 ± 0.10 ^ns^	101.6 ± 30.9 ^c^	0.76 ± 0.08 ^c^	0.64 ± 0.25 ^ab^
TU1	361.8 ± 39.9 ^c^	8.04 ± 1.2 ^b^	1.45 ±0.17 ^ab^	2.82 ±0.57 ^a^	7.92 ±0.65 ^a^	9.37 ± 0.76 ^a^	0.40 ± 0.03 ^ns^	198.7 ± 30.7 ^b^	1.19 ± 0.22 ^a^	0.53 ± 0.12 ^ab^
TU2	216.4 ± 38.7 ^d^	7.79 ± 0.7 ^b^	1.44 ±0.32 ^ab^	2.31 ±0.42 ^ab^	6.19 ±0.85 ^b^	7.63 ± 1.12 ^b^	0.30 ± 0.05 ^ns^	111.8 ± 32.3 ^c^	1.098 ± 0.09 ^bc^	0.63 ± 0.16 ^ab^
TU3	127.5 ± 16.0 ^d^	6.08 ± 0.6 ^c^	0.98 ±0.29 ^b^	1.83 ±0.16 ^b^	5.38 ± 0.21 ^b^	6.49 ± 0.09 ^b^	0.39 ± 0.15 ^ns^	54.8 ± 11.5 ^c^	1.07 ± 0.10 ^ab^	0.54 ± 0.13 ^ab^
AP2	525.1 ± 91.9 ^a^	9.06 ± 0.8 ^ab^	1.90 ±0.35 ^a^	2.69 ±0.27 ^a^	8.25 ±0.74 ^a^	10.08 ± 0.64 ^a^	0.34 ± 0.09 ^ns^	316.2 ± 38.2 ^a^	1.11 ± 0.04 ^ab^	0.72 ± 0.20 ^a^
BA1	409.9 ± 59.8 ^bc^	8.03 ± 0.4 ^b^	1.56 ±0.40 ^ab^	2.18 ±0.21 ^ab^	7.69 ±0.41 ^a^	9.25 ± 0.34 ^a^	0.35 ± 0.05 ^ns^	220.8 ± 41.5 ^b^	1.15 ± 0.06 ^ab^	0.59 ± 0.32 ^ab^
DC	470.7 ± 29.7 ^ab^	10.6 ± 0.4 ^a^	1.45 ± 0.07 ^ab^	2.05 ±0.07 ^ab^	8.05 ±0.21 a	9.50 ± 0.34 ^a^	0.27 ± 0.05 ^ns^	210.4 ± 14.1 ^b^	0.76 ± 0.01 ^c^	0.14 ± 0.0 ^b^

Different letters on the numbers in the same column indicate significant differences (*p* ≤ 0.05) between the fruits of the accessions. ns: non-significant.

**Table 3:** Seed characteristics of pomegranate genotypes. STW: total seed weight; SL: seed length; SD: seed diameter; SW: seed weight; SSI: seed shape index.

ID	STW (g)	SL (cm)	SD (cm)	SW (g)	SSI
LI1	77.57 ± 11 ^e^	0.90 ± 0.17 ^bcd^	0.71 ± 0.03 ^bc^	0.22 ± 0.01 ^c^	1.27 ± 0.06 ^a^
TU1	162.52 ± 16.04 ^bc^	0.99 ± 0.03 ^b^	0.79 ± 0.03 ^b^	0.37 ± 0.01 ^b^	1.25± 0.12 ^a^
TU2	110.31 ± 24.01 ^de^	0.81 ± 0.03 ^cd^	0.63± 0.02 ^cd^	0.25 ± 0.01 ^c^	1.29 ± 0.05 ^a^
TU3	66.90 ±9.38 ^e^	0.72 ± 0.03 ^d^	0.53 ± 0.03 ^d^	0.24± 0.01 ^c^	1.34 ± 0.05 ^a^
AP2	210.24 ± 15.23 ^ab^	1.18 ± 0.03 ^a^	1.05± 0.03 ^a^	0.51 ± 0.02 ^a^	1.12 ± 0.05 ^a^
BA1	140.77 ± 50.22 ^cd^	0.90 ± 0.04 ^bcd^	0.69± 0.06 ^bc^	0.37 ± 003 ^b^	1.31± 0.05 ^a^
DC	240.78 ± 16.52 ^a^	1.30 ± 0.05 ^a^	0.97 ± 0.04 ^a^	0.50 ± 0.01 ^a^	1.16 ± 0.09 ^a^

Different letters on the numbers in the same column indicate significant differences (*p* ≤ 0.05) between the seeds of accessions.

**Table 4:** Physicochemical and color parameters of pomegranate juices. pH; TSS: total soluble solids; TA: titratable acidity; TSS/TA ratio; L, a*, b*: color coordinates.

ID	pH	TSS	TA	TSS/TA	L	a*	b*
LI1	3.34 ± 0.02 ^d^	16.2 ± 0.3 ^b^	3.96 ± 0.19 ^a^	4.09 ± 0.3 ^b^	18.2 ± 1.1 ^e^	18.8 ± 1.1 ^b^	3.3 ± 0.9 ^b^
TU1	3.53 ± 0.02 ^c^	15.2 ± 0.2 ^bc^	2.85 ± 0.19 ^b^	5.33 ± 0.4 ^b^	28.9 ± 2.1 ^b^	29.6 ± 1.7 ^a^	11.4 ± 1.4 ^a^
TU2	3.29 ± 0.06 ^d^	13.8 ± 0.3 ^cd^	2.18 ± 0.14 ^c^	6.33 ± 0.6 ^b^	36.2 ± 1.0 ^a^	26.9 ± 3.3 ^a^	10.6 ± 1.3 ^a^
TU3	3.65 ± 0.01 ^c^	14.4 ± 0.2 ^b-d^	2.86 ± 0.18 ^b^	5.03 ± 0.2 ^b^	30.4 ± 1.5 ^ab^	18.6 ± 1.5 ^b^	9.1 ± 1.5 ^a^
AP2	4.53 ± 0.01 ^a^	14.2 ± 0.2 ^b-d^	0.50 ± 0.10 ^e^	28.27 ± 0.5 ^a^	27.01 ± 3.2 ^bc^	19.4 ± 2.1 ^b^	8.1 ± 2.2 ^b^
BA1	4.24 ± 0.02 ^b^	12.9 ± 0.4 ^e^	1.24 ± 0.10 ^d^	10.4 ± 0.3 ^a^	21.3 ± 1.1 ^de^	15.1 ± 2.0 ^b^	2.9 ± 1.2 ^b^
DC	3.08 ± 0.03 ^e^	17.6 ± 0.3 ^a^	0.37 ± 0.10 ^e^	44.5 ± 0.9 ^a^	24.2 ± 1.1 ^b-d^	14.1 ± 1.6 ^b^	2.9 ± 0.8 ^b^

Different letters on the numbers in the same column indicate significant differences (*p* ≤ 0.05) between the juice samples.

**Table 5:** Sugar contents (g L^−1^) of pomegranate accessions. Glucose, fructose, galactose, mannitol, total sugar, and ratio G/F (G, Glucose; F, Fructose).

ID	Glucose	Fructose	Galactose	Mannitol	Total Sugar	Ratio G/F
LI1	53.91 ± 1.9 ^ab^	62.67 ± 2.1 ^ab^	0.49 ± 0.04 ^ab^	1.72 ± 0.05 ^c^	118.8 ± 2.7 ^a-c^	0.86 ± 0.02 ^b^
TU1	64.40 ± 1.1 ^a^	66.88 ± 1.3 ^a^	0.98 ± 0.5 ^a^	1.37 ± 0.3 ^c^	133.6 ± 2.5 ^a^	0.96 ± 0.06 ^a^
TU2	49.33 ± 1.7 ^b^	51.78 ± 1.1 ^b^	0.37 ± 0.1 ^b^	2.60 ± 0.2 ^ab^	104.1 ± 2.7 ^bc^	0.95 ± 0.01 ^a^
TU3	47.76 ± 1.4 ^b^	49.18 ± 3.1 ^b^	n.d.	3.09 ± 0.3 ^a^	100.0 ± 2.0 ^c^	0.97 ± 0.09 ^a^
AP2	62.24 ± 1.5 ^a^	66.96 ± 1.1 ^a^	0.57 ± 0.1 ^ab^	1.81 ± 0.03 ^c^	131.6 ± 2.6 ^ab^	0.63 ± 0.05 ^d^
BA1	42.78 ± 1.3 ^b^	62.31 ± 1.1 ^ab^	0.59 ± 0.02 ^ab^	2.62 ± 0.02 ^ab^	108.3 ± 1.0 ^a–c^	0.69 ± 0.03 ^d^
DC	45.72 ± 1.2 ^b^	58.88 ± 2.1 ^ab^	0.52 ± 0.3 ^ab^	2.17 ± 0.6 ^bc^	107.2 ± 1.6 ^a–c^	0.77 ± 0.01 ^c^

Different letters on the numbers in the same column indicate significant differences (*p* ≤ 0.05) between the juice samples. n.d.—not detected.

## Data Availability

Data are contained within the article or Supplementary Materials.

## Supplementary Materials

The following supporting information can be downloaded at: https://www.mdpi.com/article/10.3390/plants13182558/s1, Table S1: Identification of GC–MS signals and their relative flavor notes; Figure S1: The score plot obtained from the PCA (F1–F6) denoting the principal components for the total parameters studied; Figure S2: Fruit of pomegranates. The images illustrate differences in some morpho-pomological traits as reported in Table 1. The fruits are displayed at the same size in the figure, and their images do not reflect their actual sizes. For the real fruit dimensions, please refer to Table 2. References [45,46,55,56,57,58,59,60,61,62,63] are cited in Supplementary Materials.
