# Supplementary material for: Exploring Italian Autochthonous Punica granatum L. Accessions: Pomological, Physicochemical, and Aromatic Investigations
Source: Plants (Basel). 2024 Sep 12;13(18):2558. doi: 10.3390/plants13182558 (PMC11434734; doi:10.3390/plants13182558)

Figure S1. Score plot obtained from the PCA (F1–F6) denoting the principal components for the total parameters studied.

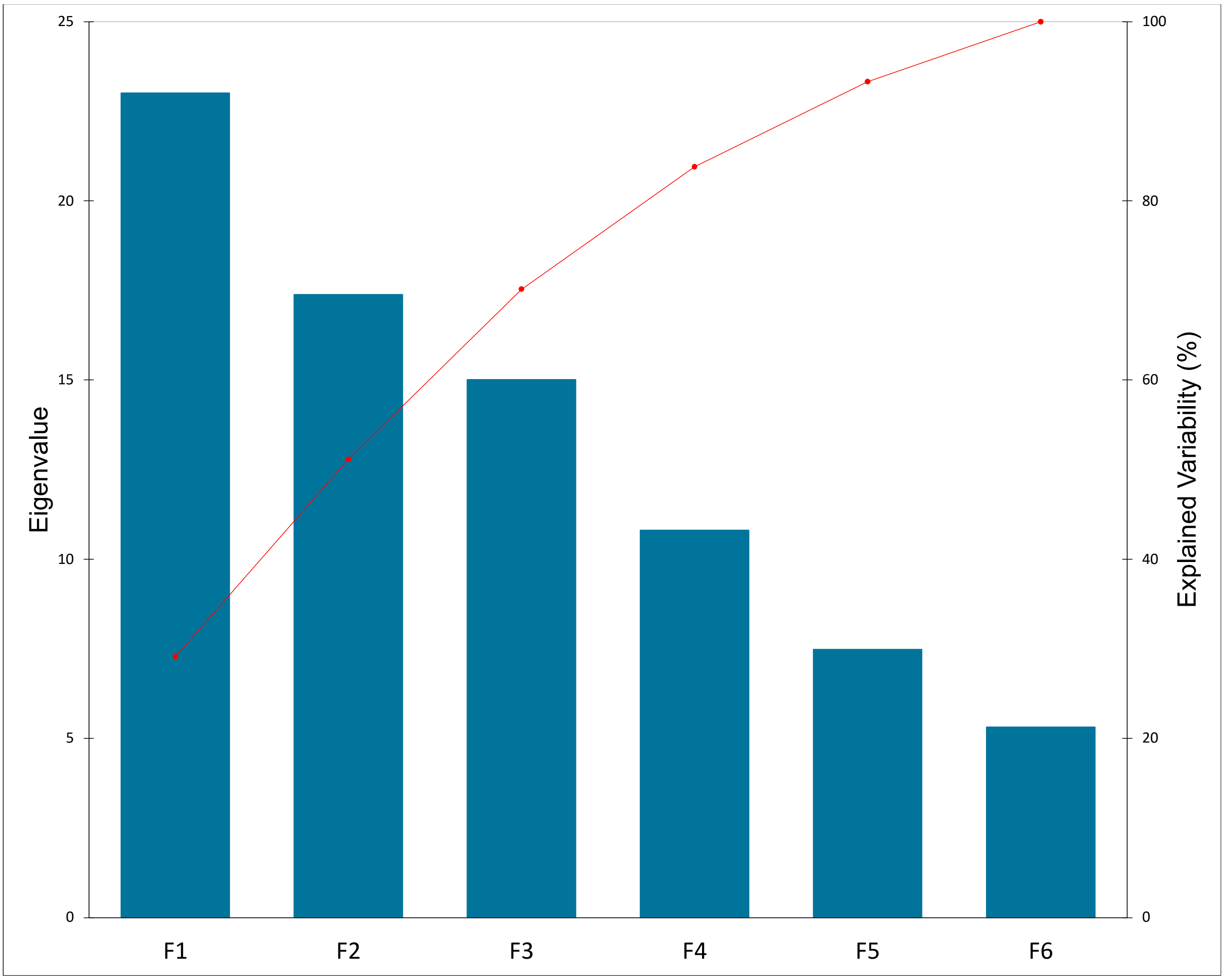

Supplement: Supplementary file 1 [file plants-13-02558-s001.zip › Figure S1-def.pdf]
