# Supplementary material for: Exploring Italian Autochthonous Punica granatum L. Accessions: Pomological, Physicochemical, and Aromatic Investigations
Source: Plants (Basel). 2024 Sep 12;13(18):2558. doi: 10.3390/plants13182558 (PMC11434734; doi:10.3390/plants13182558)

Figure S2. Fruit of pomegranates

The images illustrate differences in some morpho-pomological traits as reported in Table 1. The fruits are displayed at the same size in the figure, and their images do not reflect their actual sizes. For the real fruit dimensions, please refer to Table 2.

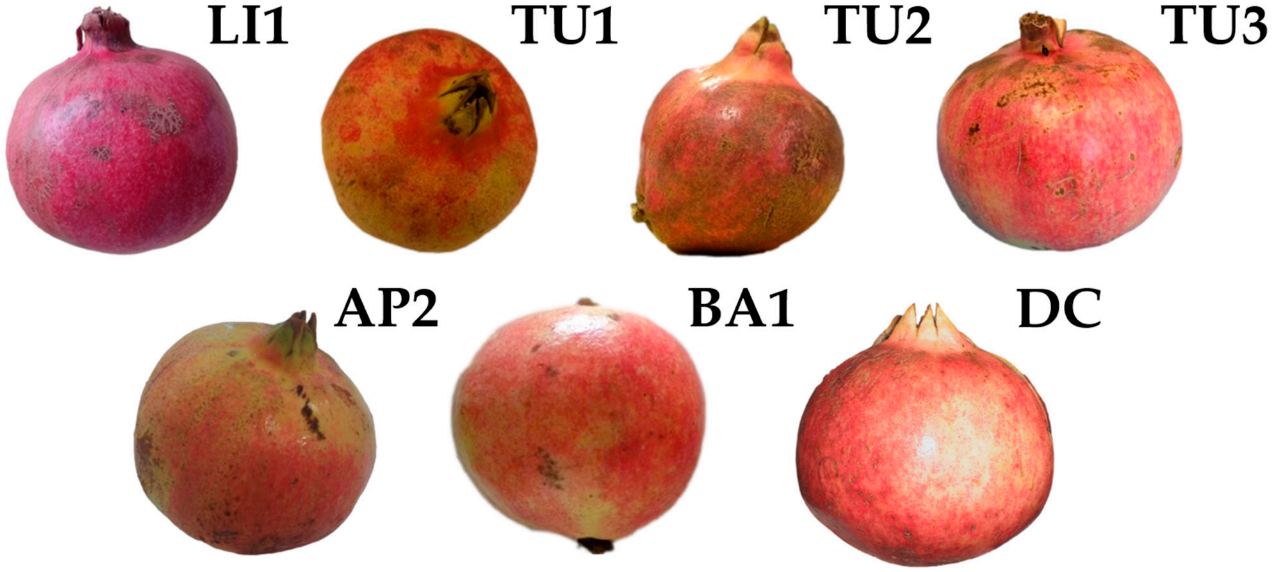

Supplement: Supplementary file 1 [file plants-13-02558-s001.zip › Figure S2-Def.pdf]
