# Supplementary material for: Exploring Italian Autochthonous Punica granatum L. Accessions: Pomological, Physicochemical, and Aromatic Investigations
Source: Plants (Basel). 2024 Sep 12;13(18):2558. doi: 10.3390/plants13182558 (PMC11434734; doi:10.3390/plants13182558)
Supplement: Supplementary file 1 [file plants-13-02558-s001.zip › Tabella S1 MDPI_IM-30Ago.pdf]

**Table S1. Volatile profile of Italian pomegranate juices for geographical origin classification.**

| Chemical class/peak number | Compound | Flavour notes [reference] | LRI | Identification method | ID* (µg/mL of pomegranate juices) | | | | | | |
|---|---|---|---|---|---|---|---|---|---|---|---|
| | | | | | LI1 | TU1 | TU2 | TU3 | AP2 | BA1 | DC |
| **Alcohols** | | | | | | | | | | | |
| 10 | Isoamyl alcohol | Alcoholic, whiskey [55] | 1233 | MS + LRI | n.d. | n.d. | n.d. | n.d. | n.d. | 1.90±0.14 | n.d. |
| 23 | 1-Hexanol | Herbal [55] | 1371 | MS + LRI | 62.72±1.93 | 228.22±17.42 | 55.01±0.3 | 63.90±0.04 | 23.63±0.28 | 100.56±8.10 | 40.87±3.15 |
| 24 | (E)-3-Hexen-1-ol | Green, leafy [55] | 1376 | MS + LRI | 0.44±0.43 | 1.28±0.21 | 0.42±0.41 | 0.72±0.03 | 0.10±0.03 | 0.57±0.19 | n.d. |
| 25 | (Z)-3-Hexen-1-ol | Green, leafy [55] | 1389 | MS + LRI | 69.20±2.80 | 50.05±3.70 | 22.47±0.81 | 44.94±3.60 | 8.42±0.07 | 14.30±0.11 | 17.51±1.14 |
| 30 | 1-Octen-3-ol | Earthy [56] | 1464 | MS + LRI | 0.57±0.06 | 0.61±0.01 | 0.29±0.28 | 0.28±0.13 | 0.17±0.04 | 0.60±0.04 | 1.07±0.19 |
| 33 | 2-Ethyl-1-hexanol | Citrus [45] | 1469 | MS + LRI | 0.86±0.06 | 2.33±0.66 | 1.74±0.72 | 1.08±0.41 | 0.39±0.03 | 1.28±0.05 | 1.28±0.38 |
| 35 | 2-Nonanol | Waxy, green, creamy [45] | 1527 | MS + LRI | 0.02±0.03 | 0.24±0.08 | n.d. | 0.06±0.08 | n.d. | n.d. | n.d. |
| 39 | 1-Octanol | Waxy, green [57] | 1573 | MS + LRI | 0.87±0.04 | 0.85±0.00 | 1.46±0.12 | 1.38±0.74 | 0.59±0.12 | 1.61±0.17 | 1.27±0.42 |
| 47 | 1-Nonanol | Fresh, fatty, floral [56] | 1675 | MS + LRI | n.d. | n.d. | n.d. | 0.85±0.06 | 0.46±0.03 | 1.07±1.06 | n.d. |
| 56 | Phenyl ethyl alcohol | Floral, rose [45] | 1887 | MS + LRI | 1.42±0.04 | 1.70±0.35 | 1.30±0.46 | 0.97±0.61 | 0.49±0.06 | 3.05±0.17 | 1.47±0.31 |
| Aldehydes | | | | | | | | | | | |
| 1 | Hexanal | Herbal [46] | 1107 | MS + LRI | 51.42±10.26 | n.d. | 17.95±0.45 | 18.35±1.16 | 11.12±0.05 | 68.54±2.92 | 101.27±15.77 |
| 5 | Heptanal | Fresh, aldehydic [58] | 1212 | MS + LRI | 0.64±0.36 | 0.74±0.10 | n.d | 0.65±0.01 | 0.13±0.01 | 0.82±0.46 | 0.49±0.09 |
| 11 | 2-Hexenal | Sweet, almond [45] | 1247 | MS + LRI | 22.32±1.36 | n.d. | 2.09±0.09 | 3.39±0.99 | 0.24±0.01 | 1.22±0.40 | 2.29±0.19 |
| 19 | Octanal | Aldehydic [55] | 1314 | MS + LRI | 1.42±0.06 | 0.50±0.05 | 0.99±0.11 | 3.13±2.29 | 0.49±0.20 | 3.15±1.56 | 2.65±1.53 |
| 21 | 2-Heptenal | Green, fatty [59] | 1351 | MS + LRI | 0.54±0.28 | n.d. | n.d. | n.d. | 0.20±0.02 | n.d. | 0.77±0.32 |
| 27 | Nonanal | Waxy [57] | 1415 | MS + LRI | 8.52±1.07 | 2.69±1.55 | 6.73±1.50 | 12.02±12.02 | 2.58±1.12 | 17.39±5.55 | 16.47±7.16 |
| 28 | 2-Octenal | Green [55] | 1452 | MS + LRI | 1.19±0.02 | n.d. | n.d. | n.d. | n.d. | n.d. | 0.75±0.42 |

| | | | | | | | | | | | |
|---|---|---|---|---|---|---|---|---|---|---|---|
| 32 | Furfural | Sweet, woody, almond [57] | 1493 | MS + LRI | 2.50±0.30 | 1.16±0.04 | 2.00±1.94 | 1.79±1.77 | 0.41±0.07 | 1.02±0.11 | 1.05±0.09 |
| 34 | Decanal | Sweet, aldehydic [60] | 1515 | MS + LRI | 1.39±0.29 | 0.80±0.70 | 3.06±0.11 | 1.68±0.61 | 0.73±0.21 | 5.49±1.38 | 2.54±0.06 |
| 36 | Benzaldehyde | Fruity [56] | 1543 | MS + LRI | 1.11±0.07 | 2.26±0.39 | 1.04±0.09 | 0.65±0.39 | 0.42±0.12 | 0.84±0.27 | 1.49±0.80 |
| 37 | 2-Nonenal | Green [56] | 1557 | MS + LRI | 1.20±0.04 | n.d. | n.d. | 0.14±0.20 | n.d. | n.d. | 0.21±0.01 |
| 40 | 5-Methylfurfural | Bready [45] | 1583 | MS + LRI | 0.48±0.23 | 0.79±0.12 | 0.89±0.14 | 0.43±0.18 | 0.50±0.12 | 0.14±0.07 | 0.57±0.11 |
| 50 | Dodecanal | Soap, waxy [57] | 1736 | MS + LRI | 0.66±0.30 | 0.58±0.22 | 0.58±0.02 | 0.32±0.05 | 0.16±0.05 | 0.55±0.12 | 0.53±0.07 |
| 57 | 2,5-Furandicarboxya-ldehyde | | 1947 | MS + LRI | 1.33±0.21 | 1.23±0.01 | 1.20±0.44 | 0.55±0.28 | 0.34±0.03 | 1.21±0.10 | 1.13±0.02 |
| Esters | | | | | | | | | | | |
| 2 | Isoamyl acetate | Sweet, fruity, banana [55] | 1141 | MS + LRI | n.d. | n.d. | n.d. | 37.34±6.27 | 7.88±0.14 | 14.42±0.55 | n.d. |
| 13 | Ethyl caproate | Sweet, fruity, pineapple [45] | 1255 | MS + LRI | n.d. | n.d. | n.d. | n.d. | 0.16±0.02 | n.d. | n.d. |
| 17 | Hexyl acetate | Fruity, green, apple [45] | 1292 | MS + LRI | 0.55±0.56 | 0.77±0.02 | n.d. | 0.59±0.00 | n.d. | n.d. | n.d. |
| 29 | Ethyl caprylate | Fruity, winey [45] | 1460 | MS + LRI | 1.16±0.60 | 1.16±0.32 | 0.70±0.34 | 0.52±0.41 | 0.37±0.03 | 0.98±0.12 | 1.69±0.87 |
| 43 | Ethyl caprate | Sweet, waxy [45] | 1622 | MS + LRI | 1.59±1.27 | 1.60±1.64 | 0.45±0.34 | 0.28±0.13 | 0.34±0.00 | 1.54±0.24 | 1.42±0.27 |
| 51 | Methyl salicylate | Wintergreen mint [55] | 1776 | MS + LRI | 0.69±0.18 | 1.32±0.23 | 0.58±0.26 | 1.47±0.63 | 0.21±0.02 | 0.84±0.22 | 1.96±0.20 |
| 53 | Ethyl laureate | Sweet, waxy [45] | 1847 | MS + LRI | 0.63±0.09 | 0.35±0.09 | 0.42±0.24 | n.d. | n.d. | n.d. | 0.41±0.07 |
| Hydrocarbons | | | | | | | | | | | |
| | p-Xylene | - | | MS | 4.42±0.44 | 0.55±0.09 | 0.37±0.11 | n.d. | n.d. | n.d. | 0.33±0.27 |
| | m-Xylene | Plastic | | MS | 1.37±0.34 | 0.29±0.31 | n.d. | n.d. | n.d. | 0.75±1.06 | 0.90±0.13 |
| | o-Xylene | Floral, geranium | | MS | 2.18±0.16 | 0.34±0.35 | 0.38±0.17 | n.d. | n.d. | n.d. | 0.35±0.16 |
| 12 | 2-Pentylfuran | Fruity [56] | 1254 | MS + LRI | n.d. | 1.10±0.07 | 0.68±0.06 | 0.32±0.14 | 0.08±0.01 | 0.36±0.04 | 0.33±0.11 |

| | | | | | | | | | | |
|---|---|---|---|---|---|---|---|---|---|---|
| 15 | Styrene | Sweet balsam, floral [55] | 1279 | MS + LRI | n.d. | 5.81±0.60 | 7.41±2.09 | 5.15±0.25 | 4.43±0.05 | 6.23±0.34 | n.d. |
| | m-Di-tert-butylbenzene | - | | MS | 1.14±0.05 | n.d. | n.d. | n.d. | n.d. | n.d. | 0.64±0.04 |
| Ketones | | | | | | | | | | | |
| 4 | 2-Heptanone | Fruity, spicy [61] | 1211 | MS + LRI | n.d. | n.d. | 2.17±1.45 | n.d. | n.d. | n.d. | n.d. |
| 9 | 4-Methyl-2-heptanone | | 1230 | MS + LRI [NIST] | 1.30±0.56 | n.d. | n.d. | n.d. | n.d. | n.d. | n.d. |
| 18 | 2-Octanone | Soap | 1308 | MS | 0.41±0.02 | 1.17±0.65 | 0.94±0.60 | n.d. | n.d. | n.d. | n.d. |
| 20 | 2,3-Octanedione | Asparagus [62] | 1347 | MS + LRI | n.d. | n.d. | n.d. | n.d. | n.d. | n.d. | n.d. |
| 26 | 2-Nonanone | Fruity [56] | 1408 | MS + LRI | 5.58±0.20 | 2.30±0.10 | 4.11±1.31 | n.d. | 0.75±0.01 | 1.13±0.25 | 0.69±0.08 |
| | 6-Methyl-5-hepten-2-one (Sulcatone) | Citrus, green | | MS | 0.79±0.08 | 1.03±0.05 | 1.24±0.38 | 0.58±0.27 | 0.26±0.04 | 0.55±0.34 | 0.87±0.12 |
| Terpenes and derivates | | | | | | | | | | | |
| 3 | β-Myrcene | Peppery, spicy [46] | 1182 | MS + LRI | 3.02±0.49 | 2.22±0.20 | 0.62±0.43 | 0.67±0.14 | 0.15±0.02 | 0.40±0.03 | 3.15±0.76 |
| 6 | Limonene | Citrus [46] | 1282 | MS + LRI | 12.72±0.35 | 8.29±0.93 | 1.03±0.13 | 6.46±1.25 | 0.96±0.07 | 17.50±1.06 | 18.71±1.82 |
| 7 | Terpene not identified | | 1216 | MS | 1.48±0.28 | 0.64±0.05 | 0.41±0.01 | n.d. | n.d. | n.d. | 5.31±0.91 |
| 8 | Eucalyptol | Eucalyptus, herbal [58] | 1230 | MS + LRI | n.d. | n.d. | n.d. | n.d. | n.d. | 3.74±0.04 | n.d. |
| 14 | γ-Terpinene | Terpenic [56] | 1265 | MS + LRI | 1.45±0.27 | 0.86±0.07 | n.d. | 0.08±0.12 | n.d. | n.d. | 1.56±0.44 |
| 16 | o-Cymene | Citrus | 1292 | MS | 2.53±0.14 | 2.08±0.06 | n.d. | 0.84±0.15 | n.d. | 2.08±0.39 | 4.29±0.32 |
| 28 | β-linalool | Floral [55] | 1566 | MS + LRI | 0.82±0.05 | 1.15±0.12 | 1.06±0.01 | 2.17±0.00 | 0.77±0.05 | 2.18±0.20 | 0.57±0.22 |
| 41 | β-Caryophyllene | Sweet, woody [46] | 1608 | MS + LRI | n.d. | 0.85±0.05 | n.d. | n.d. | n.d. | 15.78±1.48 | 12.13±2.34 |
| 42 | Terpinen-4-ol | Peppery, woody [45] | 1624 | MS + LRI | 0.85±0.20 | 0.72±0.09 | n.d. | 0.52±0.04 | 0.05±0.01 | 1.03±0.05 | 2.16±0.43 |
| 44 | Menthol | Peppermint [63] | 1666 | MS + LRI | n.d. | n.d. | 0.51±0.02 | 1.46±0.09 | n.d. | n.d. | n.d. |
| 48 | α-Terpineol | Pine, terpenic [46] | 1686 | MS + LRI | 6.87±0.20 | 8.23±0.64 | 3.36±0.02 | 2.30±0.10 | 1.17±0.01 | 5.46±0.37 | 7.51±0.94 |
| 54 | p-Cymen-8-ol | Fruity, cherry [58] | 1873 | MS + LRI | 0.37±0.36 | 0.56±0.06 | 0.38±0.12 | n.d. | n.d. | 0.37±0.11 | 0.37±0.09 |
| 55 | Geranyl acetone | Green, fruity | 1882 | MS | 1.15±0.07 | 0.72±0.03 | 1.49±0.64 | 0.51±0.32 | 0.32±0.01 | 0.90±0.13 | 1.09±0.18 |
| Other | | | | | | | | | | | |

| | | | | | | | | | | | |
|---|---|---|---|---|---|---|---|---|---|---|---|
| 45 | 4-Methyl-benzaldehyde + acetophenone (co-elution) | Fruity/Floral | 1668 | MS | 2.69±0.16 | 1.17±0.10 | n.d. | n.d. | n.d. | n.d. | 1.10±0.15 |
| | Caprylic ether | - | | MS | 0.65±0.62 | 1.09±0.08 | 0.58±0.04 | 0.92±0.46 | 0.48±0.01 | 1.58±0.30 | 0.93±0.09 |
| | Number of identified compounds | | | | 47 | 43 | 38 | 39 | 36 | 39 | 44 |
| | Total identified for chemical class | | | | LI1 | TU1 | TU2 | TU3 | AP2 | BA1 | DC |
| | Alcohols | | | | 136.08±30.16 | 285.28±24.61 | 82.67±2.78 | 102.50±6.31 | 76.48±0.30 | 124.95±13.51 | 63.47±13.34 |
| | Aldehydes | | | | 94.72±11.27 | 10.75±1.24 | 36.53±3.78 | 75.83±15.95 | 54.47±0.90 | 100.37±11.48 | 132.20±26.05 |
| | Esters | | | | 4.63±3.04 | 5.20±1.67 | 2.15±0.50 | 43.07±5.35 | 13.50±0.10 | 17.78±0.45 | 5.48±1.41 |
| | Hydrocarbons | | | | 9.12±0.66 | 8.10±1.42 | 8.84±2.43 | 3.53±0.11 | 6.19±0.06 | 7.34±0.68 | 2.56±0.72 |
| | Ketones | | | | 8.08±0.25 | 4.50±0.60 | 8.47±3.74 | 0.88±0.28 | 1.13±0.03 | 1.68±0.59 | 1.56±0.28 |
| | Terpenes and derivates | | | | 31.27±2.45 | 26.32±2.07 | 8.86±1.31 | 14.18±1.97 | 25.19±0.11 | 49.45±3.65 | 56.84±7.65 |
| | Other | | | | 3.35±1.08 | 2.26±0.01 | 0.58±0.42 | 1.36±0.45 | 1.13±0.01 | 1.58±0.30 | 2.04±0.25 |
